# Y-27632 is associated with corticosteroid-potentiated control of pulmonary remodeling and inflammation in guinea pigs with chronic allergic inflammation

**DOI:** 10.1186/s12890-015-0073-4

**Published:** 2015-08-12

**Authors:** Patricia Angeli Pigati, Renato Fraga Righetti, Samantha Souza Possa, Beatriz Saraiva Romanholo, Adriana Palmeira Dias Rodrigues, Anelize Sartori Alves dos Santos, Débora Gonçalves Xisto, Mariana Alves Antunes, Carla Máximo Prado, Edna Aparecida Leick, Milton de Arruda Martins, Patrícia Rieken Macedo Rocco, Iolanda de Fátima Lopes Calvo Tibério

**Affiliations:** Department of Medicine, School of Medicine, University of São Paulo, São Paulo, Brazil; Laboratory of Pulmonary Investigation, Carlos Chagas Filho Institute of Biophysics, Ilha do Fundão, Federal University of Rio de Janeiro, Rio de Janeiro, Brazil; Department of Medicine, Laboratory of Experimental Therapeutics, LIM-20, School of Medicine, University of São Paulo, São Paulo, Brazil; University City of São Paulo (UNICID), São Paulo, Brazil; Institute of Medical Assistance to the State Public Servant of São Paulo (IAMSPE), São Paulo, Brazil

**Keywords:** Rho-kinase, Corticosteroid, Asthma model, Y-27632, Airways, Lung mechanics, Guinea pigs

## Abstract

**Background:**

Previously, we showed that treatment with the Rho-kinase inhibitor Y-27632 was able to control airway responsiveness, inflammation, remodeling, and oxidative stress in an animal model of asthma, suggesting that this drug is beneficial in asthma. However, studies evaluating the effects of these inhibitors in conjunction with corticosteroids on chronic pulmonary inflammation have not been conducted. Therefore, we evaluated the effects of treatment with the Rho-kinase inhibitor Y-27632, with or without concurrent dexamethasone treatment, on airway and lung tissue mechanical responses, inflammation, extracellular matrix remodeling, and oxidative stress in guinea pigs with chronic allergic inflammation.

**Methods:**

The guinea pigs were subjected to seven ovalbumin or saline inhalation exposures. Treatment with Y-27632 (1 mM) and dexamethasone (2 mg/kg) started at the fifth inhalation. Seventy-two hours after the seventh inhalation, the pulmonary mechanics were evaluated and exhaled nitric oxide (E_NO_) levels were determined. The lungs were removed and histological analysis was performed using morphometry.

**Results:**

The treatment of guinea pigs with the Rho-kinase inhibitor and dexamethasone (ORC group) decreased E_NO_, the maximal mechanical responses after antigen challenge, inflammation, extracellular matrix remodeling and oxidative stress in the lungs.

This therapeutic strategy reduced the levels of collagen and IFN-γ in the airway walls, as well as IL-2, IFN-γ, 8-iso-PGF2α and NF-κB in the distal parenchyma, when compared to isolated treatment with corticosteroid or Rho-kinase inhibitor (P < 0.05) and reduced the number of TIMP-1-positive cells and eosinophils in the alveolar septa compared to corticosteroid-treated animals (P < 0.05). The combined treatment with the Rho-kinase inhibitor and the corticosteroid provided maximal control over the remodeling response and inflammation in the airways and parenchyma.

**Conclusions:**

Rho-kinase inhibition, alone or in combination with corticosteroids, can be considered a future pharmacological tool for the control of asthma.

## Background

Asthma is a complex chronic respiratory disease that depends on the interaction of genetic and environmental factors [9], and it features the activation of Th2 cells. Studies in humans and in animal asthma models have shown that inflammation, obstruction, and remodeling occur not only in the proximal airways but also in the distal pulmonary parenchyma [1, 5, 49]. The impairment of the distal airways is recognized as an important factor contributing to airflow obstruction [23], particularly in patients with severe asthma and nocturnal asthma [7].

The remodeling response in asthmatics contributes to significant changes in the structures of the proximal and distal airways [40], as well as in the extent of airflow obstruction. This process involves airway smooth muscle hypertrophy and hyperplasia, mucous gland hyperplasia, and an increase in the thickness of the airway wall [4]. Currently, anti-inflammatory therapies, such as corticosteroids, are considered the gold-standard treatment for asthma, particularly during an acute asthma attack. Corticosteroids inhibit numerous pro-inflammatory responses and induce numerous anti-inflammatory pathways. However, the development of new drugs that control this disease is essential for patients with severe, corticosteroid-insensitive asthma [18] and to decrease the collateral systemic effects of steroid use.

Under physiologic conditions, smooth muscle contraction is controlled by the phosphorylation of the myosin light chains [35] via Rho-mediated Ca^2+^ sensitization [45], or by myosin phosphatase activity, which dephosphorylates the myosin light chains [15] independently of the cytoplasmic calcium concentration [21].

The inhibition of Rho/Rho-kinase pathway may be considered a potential pharmacological and therapeutic target in lung diseases [47] because it relaxes the airway smooth muscle tone and decreases airway inflammation and remodeling. These various functions are associated with changes in the actin cytoskeleton, including cell adhesion, motility, migration, and contraction [50]. The increased activity of Rho-kinase in the vascular smooth muscle under pathophysiological conditions has been reported in hypertensive animal models [33] and in humans [26]. Thus, inhibitors of Rho/Rho-kinase that relax the airway smooth muscle and reduce muscle tone are predicted to be relevant to asthma treatment [6, 12].

Recently, our research group published promising results using the Rho-kinase inhibitor Y-27632 in an animal model of chronic allergic inflammation. Treatment with Y-27632 (a pyridine derivative, (+)-(R)-trans–4-(1-aminoethyl)-N-(4-pyridyl) cyclohexane carboxamide), a selective inhibitor of Rho-kinase family enzymes, in sensitized animals reduced lung mechanics, inflammation, remodeling and oxidative stress in the airway and lung tissue [36, 41].

Considering the relevance of corticosteroids as the gold standard treatment for asthma and aiming to complement our previous research, the focus of the present study was to evaluate the importance of combining a new class of drugs for controlling bronchial smooth muscle contraction with a corticosteroid. Knowing that the peripheral lung tissue is involved in asthma physiopathology, especially in severely asthmatic patients [13], we decided to broaden our research focus to not only include the evaluation of the airways but also study the lung parenchyma. We believe that this approach differentiates our study from those previously conducted by comprehensively covering two different compartments that determine the pathogenesis of this disease.

## Methods

### Animals

The animals weighed approximately 300–350 g initially and were approximately 3 weeks old. All guinea pigs were humanely cared for during all experimental procedures and in full compliance with the “Guide for care and use of laboratory animals” (NIH publication 85–23, revised 1985). All of the experiments described in this study were supervised and approved by the Institutional Review Board of the University of São Paulo (São Paulo, Brazil).

### The experimental model of pulmonary allergic inflammation

**Fig. 1:**
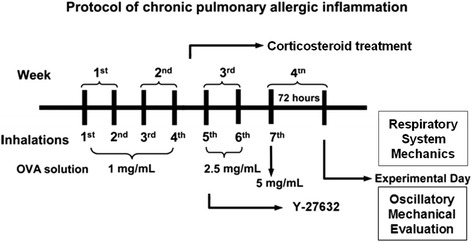
Timeline of the experimental protocol. The guinea pigs received 7 inhalation exposures (2 per week over 4 weeks) with aerosols of saline (SAL group) or ovalbumin solution (OVA group) containing an increasing dose of the antigen. From the 1st to the 4th exposures, the animals received 1 mg/mL ovalbumin (first two weeks). In the 5th and 6th exposures (third week), the animals received 2.5 mg/mL ovalbumin, and in the 7th exposure (beginning of the fourth week), a dose of 5 mg/mL of antigen was used. The solution of ovalbumin or saline was continuously aerosolized for either 15 min or until respiratory distress occurred. After the 5th exposure, the guinea pigs in the Rho-kinase inhibitor group received Y-27632 (1 mM; 2 min) by inhalation (OVA-RHO group) and/or dexamethasone (2 mg/kg-1/day i.p.) (OVA-C groups or ORC groups) 10 min before each exposure to OVA or SAL until the end of the experimental protocol. Seventy-two hours following the seventh exposure, the animals were anesthetized and exsanguinated, the exhaled nitric oxide was collected, and the mechanics of the respiratory system (Ers and Rrs) and the oscillatory parameters of the distal lung tissue (Et and Rt) were evaluated under basal conditions and after challenge with OVA (0.1 %). Afterwards, lung slices were removed and submitted to histopathological evaluation

Chronic airway inflammation was induced as previously described [1, 24, 25]. The guinea pigs were individually placed in plexiglass boxes (30 × 15 × 20 cm) coupled to an ultrasonic nebulizer (Soniclear, São Paulo, Brazil), and an aerosol of ovalbumin solution (Grade V, Sigma Chemical Co., Saint Louis, MO) diluted in 0.9 % NaCl sterile saline solution was generated for 15 min or until respiratory distress occurred. This protocol consists of a total of seven inhalation exposures. The exposures were performed twice a week for four weeks, with increasing concentrations of ovalbumin (1–5 mg/mL) to avoid tolerance (Fig. 1). For inhalations 1 to 4 (first two weeks), the dose of ovalbumin used was 1.0 mg/mL. On the fifth and sixth inhalations, animals received a solution with 2.5 mg/mL of ovalbumin, and for the seventh inhalation, the dose was increased to 5.0 mg/mL. The control animals received aerosolized saline [51].

### Treatment with Rho-kinase inhibitor

From the fifth inhalation of the experimental protocol onward, the guinea pigs were exposed for 2 min by inhalation to 1 mM Y-27632 for 10 min before each ovalbumin or normal saline exposure, as described in a previous study [17]. The authors of that study verified that a 2 min inhalation exposure with 1 mM of Y-27632 inhibited acetylcholine-induced increases in lung resistance without altering the mean blood pressure. They performed acetylcholine exposures 10 min after inhalation of Y-27632 and verified that 1 h later, the drug was already affecting lung resistance. We did not give a higher concentration of Y-27632 by intravenous injection because it can decrease the mean blood pressure, as shown in hypertensive rats [52]. In our study, on the day of the mechanical evaluation, the Y-27632 inhalation exposure was performed 1 h before the start of the experiment. We opted to administer the drug via inhalation because of the direct effect on the respiratory system and to minimize systemic effects.

### Corticosteroid treatment

To avoid interference with the OVA sensitization, immediately before the fifth inhalation the guinea pigs received dexamethasone (2 mg/.kg-1/day i.p.) five hours before ovalbumin or saline inhalation until the end of the experimental protocol. In a previous study, using the passive cutaneous anaphylaxis technique (PCA), we observed an increase in specific IgG1 homocytotropic anaphylactic antibodies in guinea pigs that received four inhalation exposures with ovalbumin at the end of the second week of the PCA protocol [25, 44].

### Experimental groups

Five groups of guinea pigs were studied:

A) animals that received normal saline (SAL, n = 8);
B) animals that received ovalbumin (OVA, n = 8);
C) animals that received ovalbumin and Y-27632 after the fifth inhalation exposure (OVA-RHO group, n = 8);
D) animals that received ovalbumin and corticosteroids (OVA-C group, n = 8);
E) animals that received ovalbumin, Y-27632 after the fifth inhalation, and corticosteroid treatment (ORC group, n = 8).

The experiment was performed in duplicate to evaluate the oscillatory distal lung mechanics.

### Measurement of E_NO_

Seventy-two hours after the last inhalation exposure, the guinea pigs were anesthetized with pentobarbital sodium (50 mg/kg, intraperitoneal injection), a tracheotomy was performed and the animals were mechanically ventilated at 60 breaths/min with a tidal volume of 8 mL/kg using a Harvard 683 ventilator (Harvard Apparatus, USA). After that, the exhaled nitric oxide (E_NO_) level was measured as previously described [25, 37]. Briefly, to obtain the E_NO_ levels after stabilization of the animal on the ventilator, a collection bag was attached to the expiratory output of the ventilator and exhaled air was collected for 3 min. E_NO_ was measured by a chemiluminescence technique using a fast-responding analyzer (NOA 280; Sievers Instruments, Boulder, CO). The analyzer was calibrated with a certified 47 parts per billion (ppb) NO source (White Martins, São Paulo, Brazil) and zero nitric oxide (NO) filter (Sievers Instruments) before each measurement. The NO filter was attached to the inspiratory input to avoid environmental contamination.

### Pulmonary mechanics evaluation

#### Respiratory system mechanics

After the E_NO_ measurement, mechanical evaluation was conducted. The tracheal pressure (Ptr) was measured using a 142PC05D differential pressure transducer (Honeywell, Freeport, IL) connected to a side tap in the tracheal cannula. Airflow (V’) was obtained using a pneumotachograph (Fleish-4-0, Richmond, VA) connected to the tracheal cannula and to a Honeywell 163PC01D36 differential pressure transducer. Lung volume (V) changes were determined by digital integration of the airflow signal. Nine to ten respiratory cycles were averaged to provide one data point. The Ptr, V’ , and V signals were collected before and after the SAL or OVA challenge and were stored in a microcomputer [37, 44, 51]. The baseline measurements of Ptr and V’ were performed after stabilization of the animal on the ventilator. Afterwards, the animals were exposed to 2 min of inhalation of either an aerosol of ovalbumin (30 mg/mL, given to the OVA and OVA-RHO groups) or normal saline (given to the SAL groups) delivered into the inspiratory circuit through the air inlet of the ventilator. Measurements of the Ptr and V’ were taken 1 and 3 min after the beginning of the first OVA/SAL challenge. Respiratory system elastance (Ers) and resistance (Rrs) were obtained using the equation of motion of the respiratory system: Ptr(t) = Ers · V(t) + Rrs · V’(t), where t represents time. Immediately after the end of the mechanical evaluation, a positive end-expiratory pressure of 5 cmH_2_O was applied and the trachea was occluded with a 5.0 silk suture at the end of the expiration to maintain lung inflation. Then, the guinea pigs were exsanguinated via the abdominal aorta, the anterior chest wall was removed, and the lungs were removed *en bloc.* The removed lungs were fixed with buffered 10 % paraformaldehyde for 24 h and then transferred to 70 % ethanol to prepare histological slides for morphometric analysis.

#### Lung oscillatory mechanics

After the last inhalation exposure (72 h), the animals were anaesthetized with pentobarbital sodium (50 mg/kg) and a tracheotomy was performed. Afterwards, the thorax was opened and the animals were exsanguinated. The lungs were removed *en bloc* and placed in a modified Krebs-Henseleit (K-H) solution (containing, in mM: 118.4 NaCl, 4.7 KCl, 1.2 K_3_PO_4_, 25 NaHCO_3_, 2.5 CaCl_2_ · H_2_O, 0.6 MgSO_4_ · H_2_O, and 11.1 glucose) at pH = 7.40 and 6 °C (63). Strips (2 × 2 × 10 mm) were cut from the periphery of the left lung and suspended vertically in a K-H organ bath that was maintained at 37 °C and continuously bubbled with a mixture of 95 % O_2_-5 % CO_2_. The lung strips were weighed, and their unloaded resting lengths (*L*_0_) were determined using a caliper. The lung strip volume was measured by simple densitometry and calculated as: volume = ΔF/δ, where ΔF is the total change in force before and after immersion of the strip in K-H solution, and δ is the mass density of K-H solution [34]. Parenchymal strips were suspended vertically in a K-H organ bath (30 mL internal volume) that was maintained at 37 °C and continuously bubbled with 95 % O_2_-5 % CO_2_, as previously described [43]. Briefly, one end of the strip was attached to a force transducer (LETICA TRI-110; Scientific Instruments, Barcelona, Spain) and the other was fastened to a lever arm actuated by means of a modified woofer, driven by a computer-generated signal and digitally converted (AT-MIO-16-E-10, National Instruments, Austin, TX). A sidearm of this rod was linked to a second force transducer (LETICA TRI-110; Scientific Instruments) by means of a silver spring of a known Young's modulus, thus allowing for the measurement of displacement. Neither amplitude dependence (<0.1 % change in stiffness) nor phase changes with frequency were detected in the 0.01- to 14-Hz range. The hysteresivity of the system (<0.003) was frequency-independent.

A cross-sectional, unstressed area (*A*_0_) of the strip was identified from the volume and the unstressed length as calculated according to *A*_0_ = vol/*L*_0_. The basal force (F_B_) for a stress of 0.1 N/cm^2^ was calculated as F_B_ (N) = 10 (N/cm^2^) × *A*_0_ (cm^2^) and adjusted by vertical displacement of the force transducer, as previously described [25]. The displacement signal was then set to zero. Once the basal force and displacement signals were adjusted, the length between bindings (*L*_B_) was measured using a precision caliper. The instantaneous length during oscillation around *L*_B_ was determined by adding the value of *L*_B_ to the measured value of displacement at any given time. The instantaneous average cross-section area (*A*_i_) was determined as *A*_i_ = vol/*L*_i_ (cm^2^), where *L*_i_ is the instantaneous length.

The instantaneous stress (σ_i_) was calculated by dividing force (F; in g) by *A*i (cm^2^) using the equation σ_i_ = F/*A*_i_. The strain was calculated as Δε = (*L* – *L*_B_)/*L*_B_. After the basal force was adjusted to 0.5 × 10^−2^ N, each parenchymal strip was preconditioned by sinusoidal oscillation of the tissue for 30 min (frequency = 1 Hz, which is a large enough amplitude to reach a final force of 1 × 10^−2^ N). Thereafter, the amplitude was adjusted to 5 % *L*_*0*_, and the oscillation was maintained for another 30 min, or until a stable length-force loop was reached. The isometric stress adaptation period resulted in a final force of 0.5 × 10^−2^ N. After preconditioning, the strips were oscillated at a frequency (*f*) of 1 Hz [42] and with a constant force of 0.5 × 10^−2^ N. The bath solution was renewed every 20 min with 37 °C K-H solution.

All mechanical parameters were measured cycle by cycle. The tissue resistance (Rt) was determined from the enclosed area of force length loops: R = (4 × *H*)/[π × ω × (Δε)^2^], where *H* is the stress–strain hysteresis area, ω is the angular frequency [ω = 2π*f* (rad/s), where *f* is the frequency], and Δε is the normalized strain or peak-to-peak change in the length divided by *L*_B_. The tissue dynamic E was determined as: Et = (Δσι/Δε)cosθ, where Δσι is the peak-to-peak change in force, and θ is the phase lag between force and displacement [(θ = sin^−1^{4 × *H*/[π(Δσι × Δε)})]. The η, which is an empirically determined variable that quantifies the dependence of dissipative processes on elastic processes, was calculated as η = tan θ. The tissue resistance (Rt), elastance (Et), and hysteresivity (η) were calculated for the baseline condition and after ovalbumin challenge (dose of 0.1 % of ovalbumin) [8].

The lung strips were fixed with buffered 10 % paraformaldehyde for 24 h and then transferred to 70 % ethanol to prepare histological slides for morphometric analysis.

### Morphometric analysis

The homogeneity of the strip samples was assured by measuring the fractional area of the tissue constituents using the point-counting method [1, 34, 53] and a 100-point grid with a known area (62,500 μm^2^ at 400× magnification) attached to the ocular of the microscope. We measured the fractional area of the bronchial wall (BW), the blood vessel wall (BVW), and the alveolar wall (AW) as the number of points that fell within BW, BVW or AW divided by the total number of points that fell within the strip tissue. The measurements were performed in 10 fields per slide at 400× magnification. We calculated the mean values for each animal.

All morphometric evaluations were performed by a blind analysis. The histologic slides were not named with the names of the groups and the researcher had no knowledge of the procedures for each group.

### Evaluation of eosinophils

Lung strips that were 5 μm thick were stained with LUNA for eosinophil evaluation [1, 41, 49]. The percentage of eosinophils in the lung parenchyma and the airway was evaluated through morphometric analysis using a reticule of known area (50 lines and 100 dots) and a microscope (CH30, Olympus, Japan). Positive cells were quantified at a magnification of 1000× using the ratio of points identifying such cells in a known area to the total number of points within the lung tissue and airway. The results are expressed in cells per unit area (10^4^ μm^2^). The quantification was performed in 10 random fields in the distal lung and three fields of the airway wall, from 3–5 airways per animal. Several authors have previously demonstrated that this method is adequate and reproducible [25, 36].

### Picro-Sirius and Resorcin-Fuchsin staining

Picro-Sirius staining was used to quantify collagen fibers and Resorcin-Fuchsin staining was used to identify elastic fibers in the airways and lung tissue. The volume fraction of collagen and elastic fibers in the airways and the alveolar tissue of the lung strips was determined (10 fields and 3–5 airways, 400×) by dividing the number of points contained within collagen or elastic fibers by the total number of points within the airway and lung tissue. The results are expressed as percentages [37, 38].

### Immunohistochemical evaluation

Immunohistochemical evaluation was performed using the following dilutions of primary antibodies: 1:400 (IL-2 – cod. sc- 7896), 1:500 (IL-4 – cod. sc-1260), 1:200 (IL-5 – cod. sc-7887), 1:200 (IL-13 – cod. 1776), 1:150 (IFN-γ – cod. sc-8308), 1:150 (NF-κB – cod. sc-109), 1:250 (MMP-9 – cod. sc-6840), 1:250 (TIMP-1 – cod. sc-5538), 1:1500 (TGF-β – cod. sc-146), all from Santa Cruz Biotechnology, Santa Cruz, CA. The anti-8-iso-prostaglandin F (PGF) 2α antibody (Oxford Biomedical Research, Rochester Hills, MI – cod. IS20) was used at a 1:500 dilution and the anti-iNOS antibody (BD Transduction Laboratories, San Diego, CA, USA, cod. N32020) was used at a 1:250 dilution. The following sequence of procedures was used to stain the samples: antigenic recovery, blockage and incubation with primary antibody, incubation with secondary antibody complex, staining and counterstaining. The slides were prepared and counts were performed in 10 random fields per sample, as described above for the evaluation of eosinophils.

### Passive cutaneous anaphylaxis reaction

To evaluate whether Rho-kinase inhibitor and/or corticosteroid treatment interfered with sensitization to ovalbumin, we measured the production of IgE and IgG1 antibodies to ovalbumin by passive cutaneous anaphylaxis (PCA), as previously described [32]. Three guinea pigs from each experimental group were anesthetized with pentobarbital sodium (50 mg/kg i.p.) on day 26 and 5 mL of blood were collected by cardiac puncture. Antibody titers were estimated by determining the highest dilution of antiserum that induced a PCA reaction. It is important to clarify that this procedure was performed one week after the seventh inhalation.

### Statistical analysis

All statistical analyses were performed using the *SigmaStat* software (SPSS Inc., USA). All data represent the means ± standard error (S.E.). The statistical significance of the differences between groups was determined using a One-Way Analysis Of Variance (ANOVA) followed by the Holm-Sidak method for multiple comparisons. We also obtained the Pearson correlation coefficient (*R*) to assess the associations of the pulmonary mechanical scores with the markers for inflammation, remodeling and oxidative stress. Differences were considered significant when P < 0.05.

## Results

### Inhalation time

**Fig. 2:**
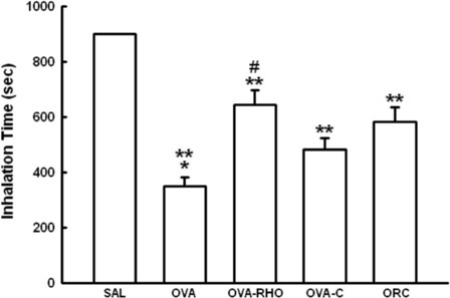
Vertical bar graph showing the mean ± SEM of the inhalation time. *P < 0.05, compared with the OVA-RHO, OVA-C and ORC groups. **P < 0.001, compared with the SAL group. ^#^P < 0.05, compared with the OVA-C group

There were no differences in the inhalation time among the groups studied until the fourth inhalation, and all of the animals reached 900 s of inhalation (Fig. 2). None of the animals in the normal saline group presented with respiratory distress during the seven inhalation exposures. From the fifth to the seventh inhalation, sensitized and non-treated animals (OVA group) presented lower inhalation times compared to the groups sensitized and treated with Y-27632, the corticosteroid or Y-27632 and the corticosteroid (OVA-RHO, OVA-C and ORC groups, respectively; P < 0.05). There was a significant difference between the OVA-RHO and OVA-C groups (P < 0.05).

### Exhaled nitric oxide

**Fig. 3:**
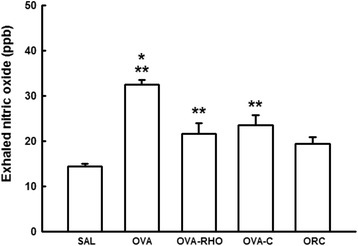
Vertical bar graph representing the mean ± SEM of the E_NO_ concentrations of the anesthetized guinea pigs. The E_NO_ was collected 72 h after the 7th exposure (before the challenge) to saline or OVA solutions in the 5 experimental groups. *P < 0.05, compared with the OVA-RHO, OVA-C and ORC groups. **P < 0.05, compared with the SAL group

The concentrations of E_NO_ were higher in the OVA group than in the SAL group (P *<* 0.001). However, treatment with Y-27632, the corticosteroid or Y-27632 and the corticosteroid reduced the level of E_NO_ compared with the OVA group (P < 0.05). There were no significance differences among the OVA-RHO, OVA-C and ORC groups (Fig. 3).

### Mechanical evaluation

#### Measurements of respiratory system mechanics

**Fig. 4:**
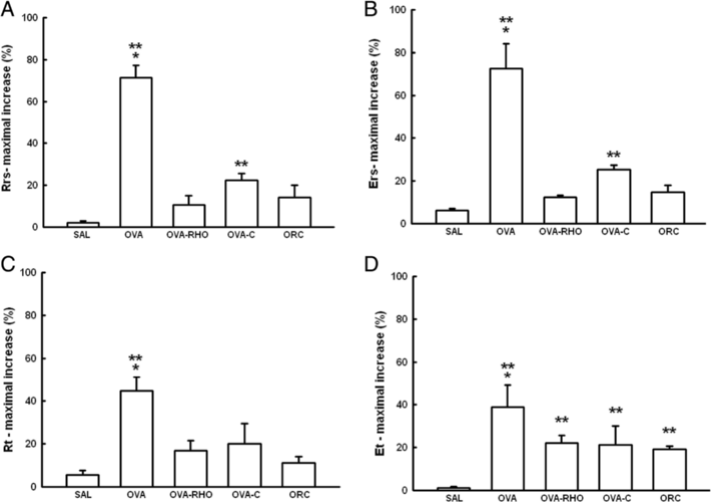
The vertical bars graph shows the mean ± SEM of the maximal percent increase in respiratory system resistance (Rrs) (**a**), respiratory system elastance (Ers) (**b**), lung tissue resistance (Rt) (**c**) and lung tissue elastance (Et) (**d**) obtained after airway and lung tissue challenge with ovalbumin or normal saline in anaesthetized guinea pigs. *P < 0.001, compared with the OVA-RHO, OVA-C and ORC groups. **P < 0.05, compared with the SAL group

Compared to the baseline values of Rrs and Ers, there were no differences among the groups (data not shown). The percentage of the maximal increase in airway Rrs is shown in Fig. 4a. There was a significant increase in the %Rrs of the OVA group compared to the control (SAL group, P < 0.001). The treatment of sensitized animals with the Rho-kinase inhibitor, the corticosteroid or both the Rho-kinase inhibitor and the corticosteroid attenuated this response (OVA-RHO, OVA-C and ORC groups, respectively) compared with the OVA group (P < 0.001).

The percentage of the maximal increase in airway Ers is shown in Fig. 4b. There was a significant increase in the %Ers of the OVA group compared to the control (SAL group, P < 0.001). The treatment of the sensitized animals with the Rho-kinase inhibitor, the corticosteroid or both the Rho-kinase inhibitor and the corticosteroid (OVA-RHO, OVA-C and ORC groups, respectively) attenuated this response compared with the OVA group (P < 0.001). There was no potentiation of this response in the ORC group.

#### Measurements of lung tissue oscillatory mechanics

Compared to the baseline values of Rt and Et, there were no differences among the groups (data not shown). The percentage of the maximal increase in airway Rt is shown in Fig. 4c. There was a significant increase in the %Rt of the OVA group compared to the control (SAL group, P < 0.001). The treatment of the sensitized animals with the Rho-kinase inhibitor, the corticosteroid or both the Rho-kinase inhibitor and the corticosteroid (OVA-RHO, OVA-C and ORC groups, respectively) attenuated this response compared with the OVA group (P < 0.05).

The percentage of the maximal increase in airway %Et is shown in Fig. 4d. There was a significant increase in the %Et of the OVA group compared to the control (SAL group, P < 0.001). The treatment of sensitized animals with the Rho-kinase inhibitor, the corticosteroid or both the Rho-kinase inhibitor and the corticosteroid (OVA-RHO, OVA-C and ORC groups, respectively) attenuated this response compared with the OVA group (P < 0.05).

There were no differences in the maximal responses in %Et and %Rt among the OVA-RHO, OVA-C and ORC groups.

### Morphometric analysis

All of the groups showed a similar anatomical composition of the lung strips, with approximately 90 % of the strip being the alveolar wall (SAL group: 93.2 ± 1.10 %, OVA group: 92.3 ± 0.76 %, OVA-RHO group: 92.6 ± 1.29 %, OVA-C group: 93.4 ± 0.85 and ORC group: 98.0 ± 0.68 %)**.**

#### Effects on inflammation

The absolute values for the number of eosinophils and the cellular inflammation markers, IFN-γ, IL-2, IL-4, IL-5 and IL-13, present in the four experimental groups are shown in Table 1 (airway and lung tissue).

**Table 1:** Absolute values of the morphometric analysis for inflammatory markers in the airway and lung tissue

AIRWAY					
BIOMARKERS	SAL (n = 8)	OVA (n = 8)	OVA-RHO (n = 8)	OVA-C (n = 8)	ORC (n = 8)
Eosinophils	4.15 ± 1.26	33.56 ± 4.66 ***	12.17 ± 1.57 */ ^#^	16.83 ± 4.34 *	11.46 ± 3.74 *
IFN-γ	9.37 ± 0.97	35.36 ± 2.50 ***	17.98 ± 1.14 ^+^	18.02 ± 0.83 ^+^	12.48 ± 1.44 ^+ / **^
IL-2	2.39 ± 0.57	32.76 ± 6.95 ***	13.31 ± 0.62 ^+^	12.47 ± 0.97 ^+^	10.09 ± 1.29 ^+^
IL-4	4.69 ± 0.68	13.69 ± 1.26 ***	6.34 ± 1.58 ^+^	8.13 ± 1.24 ^+^	5.61 ± 0.85 ^+^
IL-5	4.53 ± 0.75	21.76 ± 1.23 ***	11.43 ± 1.74 *	11.65 ± 2.12 *	9.67 ± 1.34 *
IL-13	1.19 ± 0.75	13.42 ± 0.45 ***	4.37 ± 1.12 *	6.64 ± 1.58 *	4.59 ± 0.68 *
LUNG TISSUE					
BIOMARKERS	SAL (n = 8)	OVA (n = 8)	OVA-RHO (n = 8)	OVA-C (n = 8)	ORC (n = 8)
Eosinophils	0.75 ± 0.27	11.48 ± 0.75 ***	4.13 ± 0.15 */ ^#^	6.15 ± 2.19 *	2.73 ± 1.22 */ ^#^
IFN-γ	11.26 ± 1.47	25.48 ± 1.13 ***	16.27 ± 0.76 ^+^	19.08 ± 0.61 ^+^	13.81 ± 0.78 ^+ / **^
IL-2	2.79 ± 0.49	19.53 ± 1.39 ***	7.48 ± 0.62 ^+^	9.26 ± 0.45 ^+^	4.13 ± 0.60 ^+ / **^
IL-4	2.57 ± 0.37	18.78 ± 1.53 ***	11.26 ± 0.92 ^+^	8.32 ± 0.40 ^+^	6.89 ± 0.96 ^+^
IL-5	2.25 ± 0.54	13.52 ± 0.66 ***	5.76 ± 0.47 *	7.04 ± 0.93 *	5.90 ± 1.00 *
IL-13	1.38 ± 0.59	11.37 ± 2.26 ***	4.74 ± 1.32 *	3.61 ± 1.07 *	2.62 ± 0.76 *

The eosinophils, IFN-γ, IL-2, IL-4, IL-5, IL-13, are expressed in positive cells/10^4^ μm^2^. ***P < 0.001, compared with the SAL group; *P < 0.05, compared with the OVA group; ^#^P < 0.05, compared with the OVA-C group, ^+^P < 0.001, compared with the OVA group, **P < 0.05, compared with the OVA-RHO and OVA-C groups

In the airways and lung tissue, there was an increase in the eosinophil density in the OVA group compared with the SAL group (P < 0.001). The OVA-RHO, OVA-C and ORC groups had a reduced eosinophil density in the airways and lung tissue compared with the OVA group (P < 0.05). In the lung tissue of the OVA-RHO and ORC groups, there was a reduction in the eosinophil density compared with the OVA-C group, (P < 0.05).

We observed an increase in IFN-γ-positive cells in the airways and lung tissue in the OVA group compared with the SAL group (P < 0.001). The OVA-RHO, OVA-C and ORC groups had a reduced number of IFN-γ-positive cells in the airways and lung tissue compared with the OVA group (P < 0.001). There was a reduction in the number of IFN-γ-positive cells in the airways and lung tissue of the ORC group compared with the OVA-RHO and OVA-C groups (P < 0.05).

There was an increase in the number of IL-2-positive cells in the airways and lung tissue from the OVA group compared with the SAL group (P < 0.001). The OVA-RHO, OVA-C and ORC groups had a reduced numbers of IL-2-positive cells in the airways and lung tissue compared with the OVA group (P < 0.001). Notably, there was also a significant decrease in the number of IL-2-positive cells in the lung tissue from the ORC group compared to tissue from the OVA-RHO and OVA-C groups (P < 0.05).

There was an increase in the number of IL-4-positive cells in the airways and lung tissue from the OVA group compared with the SAL group (P < 0.001). The OVA-RHO, OVA-C and ORC groups had reduced numbers of IL-4-positive cells in the airways and lung tissue compared with the OVA group (P < 0.001).

There was an increase in the numbers of IL-5- and IL-13-positive cells in the airways and lung tissue from the OVA group compared with the SAL group (P < 0.001). The OVA-RHO, OVA-C and ORC groups had reduced numbers of IL-5- and IL-13-positive cells in the airways and lung tissue compared with the OVA group (P < 0.05).

**Fig. 5:**
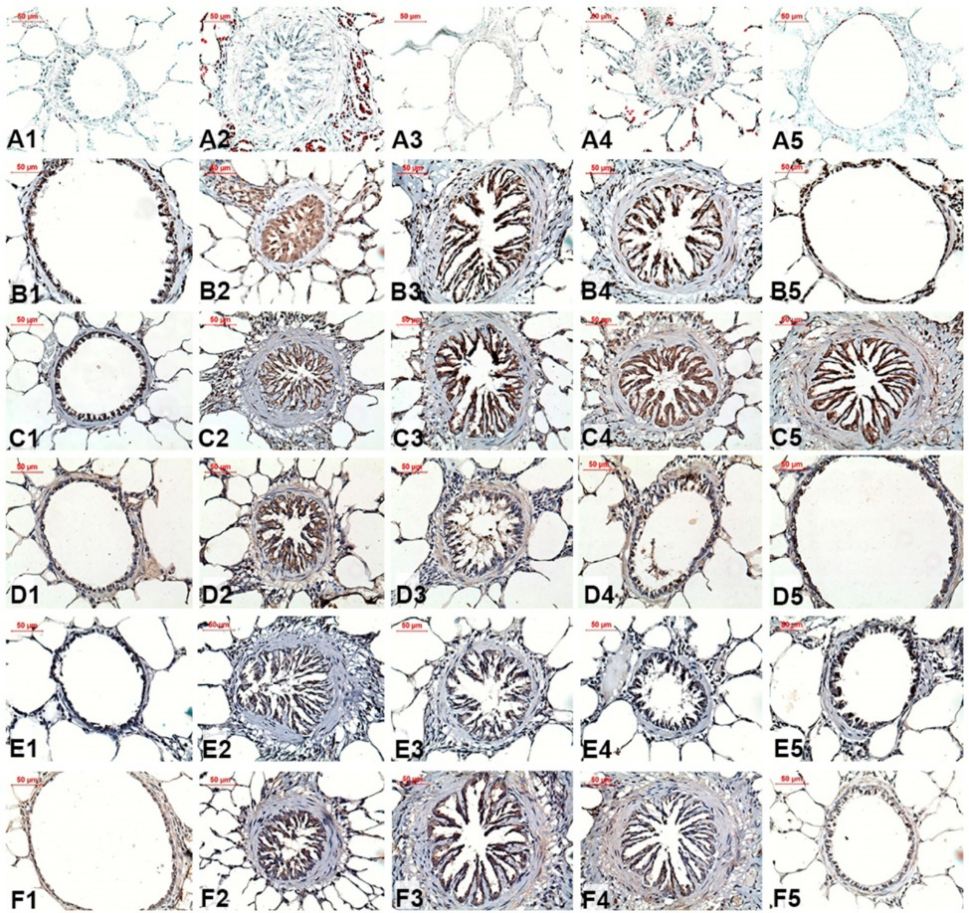
Photomicrographs of the immunohistochemical analyses of the airway walls to detect eosinophils (A1-A5, X400); IFN-γ (B1-B5, X400), IL-2 (C1-C5, X400), IL-4 (D1-D5, X400), IL-5 (E1-E5, X400) and IL-13 (F1-F5, X400) in guinea pigs exposed to saline (**a1**, **b1**, **c1**, **d1**, **e1** and **f1**) or ovalbumin (**a2**, **b2**, **c2**, **d2**, **e2** and **f2**). The treated guinea pigs exposed to Y-27632 (**a3**, **b3**, **c3**, **d3**, **e3** and **f3**), to corticosteroid (**a4**, **b4**, **c4**, **d4**, **e4** and **f4**) or to Y-27632 combined with corticosteroid (**a5**, **b5**, **c5**, **d5**, **e5** and **f5**) are also shown

Figure 5 presents photomicrographs of the airway walls from guinea pigs in the experimental groups subjected to LUNA staining for the detection of eosinophil density and immunohistochemical staining for IFN-γ, IL-2, IL-4, IL-5 and IL-13 detection.

#### Effects on extracellular matrix remodeling

The absolute values of the remodeling markers for the volume fraction of collagen, elastic fibers and actin, TGF-β, MMP-9 and TIMP-1-positive cells in the four experimental groups are shown in Table 2 (airway and lung tissue).

**Table 2:** Absolute values of the morphometric analysis for remodeling markers in the airway and lung tissue

AIRWAY					
BIOMARKERS	SAL (n = 8)	OVA (n = 8)	OVA-RHO (n = 8)	OVA-C (n = 8)	ORC (n = 8)
Collagen Fibers	9.13 ± 0.36	25.54 ± 1.95 ***	15.95 ± 0.94 *	19.60 ± 1.89 *	10.31 ± 0.97 * / **
Elastic Fibers	2.26 ± 0.43	17.68 ± 2.47 ***	4.53 ± 0.59 *	9.33 ± 0.67 *	4.87 ± 0.52 *
Actin	6.94 ± 0.67	25.59 ± 2.26 ***	12.16 ± 1.25 *	19.97 ± 0.94 *	15.30 ± 1.23 *
TGF-β	4.48 ± 0.53	13.47 ± 1.74 ***	5.35 ± 1.78 *	7.73 ± 1.58 *	5.67 ± 0.91 *
MMP-9	12.35 ± 0.78	26.48 ± 1.48 ***	11.29 ± 1.38 ^+^	13.36 ± 0.49 ^+^	10.82 ± 1.34 ^+^
TIMP-1	4.63 ± 1.25	17.17 ± 1.36 ***	8.13 ± 0.73 *	9.82 ± 1.27 *	7.30 ± 0.98 *
LUNG TISSUE					
BIOMARKERS	SAL (n = 8)	OVA (n = 8)	OVA-RHO (n = 8)	OVA-C (n = 8)	ORC (n = 8)
Collagen Fibers	8.62 ± 0.28	22.42 ± 0.68 ***	9.74 ± 0.64 *	13.76 ± 0.32 *	12.35 ± 1.73 *
Elastic Fibers	7.74 ± 0.69	19.49 ± 0.52 ***	12.93 ± 1.37 *	12.85 ± 0.51 *	8.05 ± 1.59 *
Actin	4.52 ± 0.98	13.24 ± 0.74 ***	4.65 ± 0.85 *	4.86 ± 0.84 *	4.94 ± 0.90 *
TGF-β	5.90 ± 0.63	15.24 ± 0.36 ***	6.37 ± 0.69 *	6.46 ± 1.13 *	5.69 ± 0.74 *
MMP-9	12.53 ± 0.95	27.17 ± 0.35 ***	14.53 ± 0.55 ^+^	14.92 ± 0.81 ^+^	13.55 ± 0.56 ^+^
TIMP-1	3.95 ± 0.74	17.19 ± 1.12 ***	12.58 ± 0.59 *	12.18 ± 1.27 *	8.32 ± 1.12 * / **

The collagen fibers, elastic fibers and actin are expressed in percentage of increase (%).The TGF-β, MMP-9 and TIMP-1 are expressed in positive cells/10^4^ μm^2^. ***P < 0.001, compared with the SAL group; *P < 0.05, compared with the OVA group; ^#^P < 0.05, compared with the OVA-C group, ^+^P < 0.001, compared with the OVA group, **P < 0.05, compared with the OVA-RHO and OVA-C groups

We observed an increase in the volume fraction of collagen fibers in the airways and lung tissue from the OVA group compared to the SAL group (P < 0.001). The OVA-RHO, OVA-C and ORC groups showed a reduction in the volume fraction of collagen fibers in the airways and lung tissue compared to the OVA group (P < 0.05). There was also a reduction of the volume fraction of collagen fibers in the airways of the ORC group compared to the OVA-RHO and OVA-C groups (P < 0.05).

There was an increase in the volume fraction of elastic fibers and actin in the airways and lung tissue from the OVA group compared with the SAL group (P < 0.001). The OVA-RHO, OVA-C and ORC groups had a reduced volume fraction of elastic fibers and actin in the airways and lung tissue compared with the OVA group (P < 0.05). There were no differences among the OVA-RHO, OVA-C and ORC groups (P < 0.05).

There was an increase in the number of TGF-β-positive cells in the airways and lung tissue from the OVA group compared with the SAL group (P < 0.001). The OVA-RHO, OVA-C and ORC groups had reduced numbers of TGF-β-positive cells in the airways and lung tissue compared with the OVA group (P < 0.05).

The number of MMP-9-positive cells is shown in Table 2. There was an increase in the number of MMP-9-positive cells in the airways and lung tissue from the OVA group compared with the SAL group (P < 0.001). The OVA-RHO, OVA-C and ORC groups had reduced numbers of MMP-9-positive cells in the airways and lung tissue compared with the OVA group (P < 0.001).

There was an increase in the number of TIMP-1-positive cells in the airways and lung tissue from the OVA group compared with the SAL group (P < 0.05). The OVA-RHO, OVA-C and ORC groups had reduced numbers of TIMP-1-positive cells in the airways and lung tissue compared with the OVA group (P < 0.05). There was a reduction in the number of TIMP-1 positive cells in the ORC group compared to the OVA-RHO and OVA-C groups, but only in the lung tissue (P < 0.05).

**Fig. 6:**
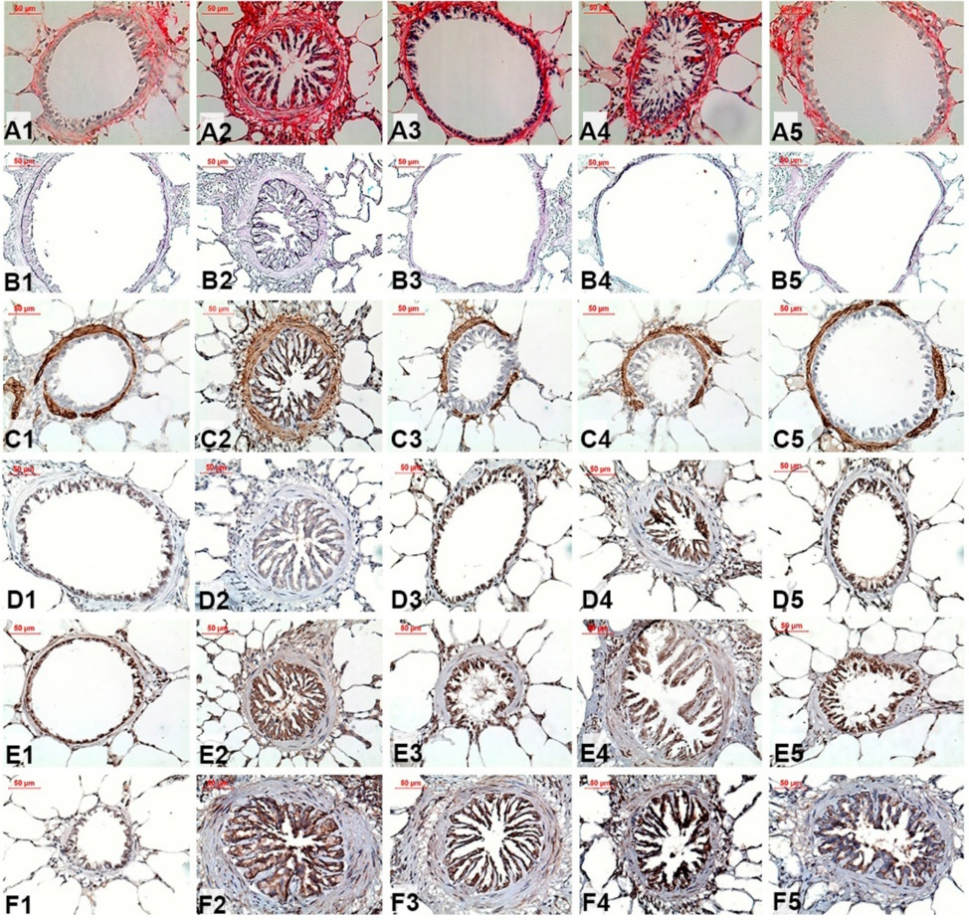
Representative photomicrographs of guinea pig airways stained with Picrosirius for collagen content (A1-A5, X400), Weighert Resorcin-Fucsin for elastic fiber content (B1-B5, X400), actin (C1-C5, X400), TGF-β (D1-D5, X400), MMP-9 (E1-E5, X400) and TIMP-1 (F1-F5, X400) in guinea pigs exposed to saline (**a1**, **b1**, **c1**, **d1**, **e1** and **f1**) or ovalbumin (**a2**, **b2**, **c2**, **d2**, **e2** and **f2**). The treated guinea pigs exposed to Y-27632 (**a3**, **b3**, **c3**, **d3**, **e3** and **f3**), to corticosteroid (**a4**, **b4**, **c4**, **d4**, **e4** and **f4**) Y-27632 combined with corticosteroid (**a5**, **b5**, **c5**, **d5**, **e5** and **f5**) are also shown

Representative photomicrographs of the airways of the animals stained with Picrosirius for collagen content, Weighert Resorcin-Fuchsin for elastic fiber content, and immunohistochemical staining for actin, TGF-β, MMP-9, TIMP-1 detection are shown in Fig. 6.

#### Effects on oxidative stress and NF-ĸB expression

The absolute values of the oxidative stress and nuclear factor markers, including the number of iNOS-positive cells, the volume fraction of 8-iso-PGF2α and the number of NF-κB-positive cells in the four experimental groups, are shown in Table 3 (airway and lung tissue).

**Table 3:** Absolute values of the morphometric analysis for oxidative stress and transcription factor markers in the airway and lung tissue

AIRWAY					
BIOMARKERS	SAL (n = 8)	OVA (n = 8)	OVA-RHO (n = 8)	OVA-C (n = 8)	ORC (n = 8)
iNOS	5.46 ± 0.17	17.44 ± 1.47 ***	7.47 ± 0.85 *	10.20 ± 2.12 *	9.70 ± 0.81 *
8-iso-PGF2α	11.25 ± 0.38	26.47 ± 1.28 ***	14.48 ± 2.97 *	15.26 ± 1.37 *	13.28 ± 1.16 *
NF-κB	6.18 ± 0.48	17.36 ± 1.46 ***	7.69 ± 0.42 *	7.29 ± 1.34 *	5.35 ± 0.89 *
LUNG TISSUE					
BIOMARKERS	SAL (n = 8)	OVA (n = 8)	OVA-RHO (n = 8)	OVA-C (n = 8)	ORC (n = 8)
iNOS	1.26 ± 0.37	10.14 ± 0.38 ***	4.37 ± 0.85 *	5.42 ± 0.94 *	4.67 ± 0.37 *
8-iso-PGF2α	1.15 ± 0.38	18.84 ± 1.85 ***	9.48 ± 0.85 *	10.14 ± 0.73 *	2.18 ± 0.54 * / **
NF-κB	1.38 ± 0.31	18.37 ± 1.78 ***	11.37 ± 1.63 *	10.88 ± 0.96 *	5.70 ± 0.94 * / **

The iNOS and NF-κB are expressed in positive cells/10^4^ μm^2^. The 8-iso-PGF2α is expressed in percentage of increase (%). ***P < 0.001, compared with the SAL group; *P < 0.05, compared with the OVA group; **P < 0.05, compared with the OVA-RHO and OVA-C groups

The number of iNOS-positive cells in the airway and lung tissue was significantly higher in the OVA group compared with the SAL group (P < 0.001). The OVA-RHO, OVA-C and ORC groups had reduced numbers of iNOS-positive cells in the airways and lung tissue compared with the OVA group (P < 0.05).

The volume fraction of 8-iso-PGF2α in the airway and lung tissue was significantly higher in the OVA group compared with the SAL group (P < 0.001). The OVA-RHO, OVA-C and ORC groups had a reduction in the volume fraction of 8-iso-PGF2α in the airways and lung tissue compared with the OVA group (P < 0.05). There was also a reduction in 8-iso-PGF2α in the ORC group compared with the OVA-RHO and OVA-C groups, but only in the lung tissue (P < 0.05).

The number of NF-κB-positive cells in the airway and lung tissue was significantly higher in the OVA group compared with the SAL group (P < 0.001). The OVA-RHO, OVA-C and ORC groups had reduced numbers of NF-κB-positive cells in the airways and lung tissue compared with the OVA group (P < 0.05). There was also a reduction in NF-κB-positive cells in the ORC group compared with the OVA-RHO and OVA-C groups, but only in the lung tissue (P < 0.05).

**Fig. 7:**
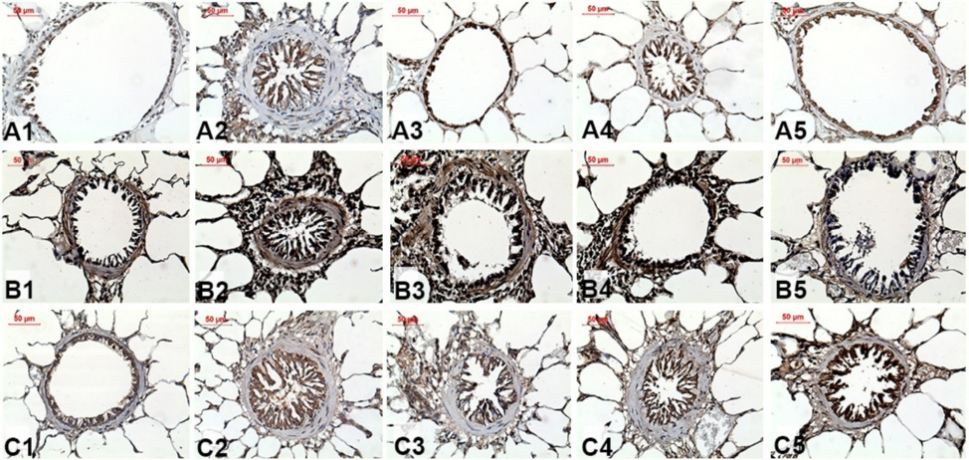
Representative photomicrographs of guinea pig airways stained with immunohistochemistry results for iNOS (A1-A5, X400), 8-iso-PGF2α (B1-B5, X400) and NF-κB (C1-C5, X400) in guinea pigs exposed to saline (**a1**, **b1** and **c1**) or ovalbumin (**a2**, **b2** and **c2**). The treated guinea pigs exposed to Y-27632 (**a3**, **b3** and **c3**), to corticosteroid (**a4**, **b4** and **c4**) or to Y-27632 combined with corticosteroid (**a5**, **b5** and **c5**) are also shown

Representative photomicrographs of the immunohistochemical staining for iNOS, 8-iso-PGF2α and NF-κB in the airways of the all experimental groups are shown in Fig. 7.

### Passive Cutaneous Anaphylaxis (PCA)

The specific antibody titration of IgG1 in the OVA animals was 1:640. However, the sensitized animals treated with the corticosteroid, the Rho-kinase inhibitor or both had lower levels of the specific IgG1 in the OVA-RHO group (1:160), the OVA-C group (1:160) and the ORC group (1:20). None of the groups had a PCA reaction to the specific IgE at any dilution of antiserum.

## Discussion

In the present study, we evaluated the effects of treatment with the Rho-kinase inhibitor Y-27632, the corticosteroid dexamethasone, or both, on guinea pigs with chronic allergic inflammation. Our analysis focused on the mechanical responses, inflammation, remodeling, and production of oxidative stress in the airways and lung parenchyma. The final results showed that both of the individual treatments were effective in reducing the maximum resistance and elastance of the respiratory system and lung parenchyma when challenged with antigen, suggesting that Rho-kinase inhibition or corticosteroid treatment modulate the constriction of the airways and lung parenchyma. There was also a reduction in E_NO_, eosinophilic infiltration, Th1/Th2 inflammatory cytokines, extracellular matrix remodeling, actin content, the number of NF-ĸB-positive cells and oxidative stress.

The combination of the Rho-kinase inhibitor with the corticosteroid decreased all of the functional and histological parameters to the same extent as the individual treatments, in addition to maximizing the reduction of collagen and IFN-γ in the airways, the number of cells positive for IL-2, IFN-γ, NF-ĸB and the volume fraction of 8-iso-PGF2α in the distal lung tissue.

In the lung parenchyma, the combined treatment of Y-27632 with the corticosteroid maximized the reduction in the eosinophil counts and TIMP-1-positive cells when compared to animals treated only with the corticosteroid.

Notably, no previous studies have examined repeated treatment with a Rho-kinase inhibitor combined with a corticosteroid as a therapeutic strategy for the control of hyperresponsiveness, remodeling and inflammatory alterations induced by chronic allergic inflammation in the airways and distal lung tissue.

To avoid interfering with the animals’ sensitization, we started treatment with the corticosteroid and/or Y-27632 twenty-four hours after the fourth ovalbumin inhalation exposure. Using the PCA technique, we found that the sensitized animals had an increase in specific IgG1 anaphylactic antibodies.

The inhalation time was recorded to evaluate the acute responses to antigen exposure, as previously described [51]. Treatment with the Rho-kinase inhibitor, the corticosteroid or the combination of these therapies attenuated the acute response in sensitized animals. The use of Y-27632 alone increased the contact time with the antigen when compared to the corticosteroid-treated group in sensitized animals. This most likely occurred because of the muscle-relaxing action of the Rho-kinase inhibitor (Y-27632) on the airway smooth muscle [46] and on the myocontractile elements of the lung parenchyma, resulting in diminished signs of respiratory distress and increased inhalation time. On the other hand, dexamethasone affects mainly the inflammatory component of asthma.

We believe that several mechanisms contributing to the acute responses to antigen challenge, including smooth muscle contraction, were most likely altered by treatment with Y-27632 and the protective effect of the corticosteroid [25, 36].

As a confirmation that these three treatments (separate use of Y-27632 and the corticosteroid, and the combined use of the corticosteroid with Y-27632) can effectively control the inflammatory process, we observed a significant reduction in E_NO_ among the animals sensitized with ovalbumin.

The E_NO_ level has been proposed as an indirect marker of lung inflammation and correlates with the severity and response to the treatments. It is also elevated in asthmatics and animal models of chronic pulmonary inflammation [2, 25, 36]. Nitric oxide is involved in various mechanisms of asthma physiopathology. Previous studies have demonstrated that nitric oxide derived from constitutive isoforms has protective effects on bronchoconstriction and remodeling [37], whereas nitric oxide produced by iNOS is involved in the constriction, inflammation and remodeling processes [38, 49].

The maximum elastance and resistance responses after antigen challenge decreased in both the respiratory system and the distal pulmonary tissue when the sensitized animals were treated with Y-27632, the corticosteroid, or a combination of both treatments, compared to the animals that received the vehicle. To further investigate the mechanisms involved in the control of the mechanical responses, we evaluated the volume fraction of actin in the airways and in the lung parenchyma of sensitized and treated guinea pigs. There was a decrease in the volume fraction of actin in the airway walls and alveolar septa in the treated groups compared to the untreated groups.

The results from different studies using the same model of experimental asthma have shown that treatment with Y-27632 is effective in decreasing the actin content in the airways [36] and in the distal parenchyma [41], but none of the studies assessed the effects of this inhibitor combined with corticosteroids. In the current study, the Rho-kinase inhibitor, alone or in combination with a corticosteroid, was able to reduce the volume fraction of actin compared to the OVA group.

There is evidence that activated Rho-kinase regulates actin/myosin contractibility regardless of the level of free calcium [31]. Actin is part of the cytoskeleton of endothelial cells, and the presence of inflammatory agonists increases cytosolic calcium levels. Increased calcium levels diminish cAMP and activate RhoA/Rho-kinase, causing the reorganization of the cortical actin into stress fibers, which are bundles of actin/myosin necessary to induce cell contraction [39]. In the distal parenchyma, the actin is localized in the myocontractile elements, such as myofibroblasts.

Corticosteroids may directly or indirectly modulate the contraction of the airway smooth muscle by suppressing the agonistic responses induced by an increase in intracellular calcium or by down-regulation linked to uncoupling receptors (M_2_ or M_3_ muscarinic, H_1_ histaminic receptors). In addition, corticosteroids can enhance the relaxation of the airway smooth muscle by activating AMP cycle-dependent or -independent mechanisms. The effects of dexamethasone on human airway smooth muscle were studied by Goldsmith et al. [10]. These authors showed that in human bronchial cells, corticosteroids reduced the level of smooth muscle-α and that such effects were mediated, at least in part, by the attenuation of mRNA translation and increased protein degradation.

The combined treatments resulted in a reduction in the number of cells positive for IFN-γ in the airways and in the lung parenchyma. We also observed a greater reduction in eosinophils and IL-2 in the lung parenchyma when both treatments were combined. Additionally, our results showed that in the lung parenchyma, treatment with the Rho-kinase inhibitor had a greater impact on reducing the eosinophil counts than treatment with the corticosteroid, showing that use of this drug by itself is promising.

In a related study, Souza et al. [48] investigated the effects of the corticosteroid montelukast and 1400 W (a selective iNOS inhibitor) in the peripheral lung tissue of guinea pigs with chronic allergic pulmonary inflammation. The results showed that the isolated use of dexamethasone was able to reduce the eosinophilic infiltrates and the number of Th1 (IFN-γ) and Th2 (IL-4 and IL-5) cells. Hence, the combination of an iNOS inhibitor and montelukast caused a maximized reduction in IL-4, IL-5, and IFN-γ. In mice chronically exposed to ovalbumin, chronic administration of budesonide reduced airway hyperresponsiveness, as well as leukocyte infiltration, with a decrease in the production of Th2 mediators such as IL-4, IL-12, and eotaxin-1 [28].

The results of the present study corroborated data in the literature describing the effect of a Rho-kinase inhibitor on eosinophil recruitment. Taki et al. [50] observed that the Rho-kinase inhibitor Fasudil reduced the number of eosinophils after allergen challenge, although it did not affect the recruitment of other inflammatory cells. Hashimoto et al. [14] demonstrated that the Rho-kinase inhibitor Y-27632 had a key role in reducing the infiltration and activation of inflammatory cells.

When we evaluated the alterations of the extracellular matrix, particularly the volume fraction of collagen and elastic fibers and the numbers of TIMP-1, MMP-9 and TGF-ß positive cells, we observed a reduction in the airway and distal lung parenchyma of the sensitized animals treated with the Rho-kinase inhibitor or with the corticosteroid. The combination of the corticosteroid with Y-27632 enhanced the reduction in the volume fraction of collagen fibers present in the airways. We also observed a greater attenuation of TIMP-1-positive cells in the lung parenchyma when both drugs were combined.

These results can be explained, at least in part, by the reduction in eosinophil recruitment and Th1/Th2 inflammatory cytokine levels, as demonstrated by the correlation data.

Studies suggest the importance of Rho-kinase modulation in the remodeling process. Zhou et al. [54] showed that Rho-kinase activation is crucial to collagen synthesis, which may be related to a combination of factors including the inhibition of the c-Jun N-terminal kinase (JNK) and TGF-ß pathways. Additionally, Kondrikov et al. [22] concluded that oxygen toxicity induces ROS to separate the guanine nucleotide dissociation inhibitor (GDI, a regulator of Rho GTPase activity) from Rho-kinase, leading to the activation of the Rho-kinase pathway and contributing to an increase in type I collagen synthesis.

Other studies suggest that inhaled corticosteroids are unable to reduce the pulmonary remodeling responses completely [16, 48]. In this regard, Goleva et al. [11] showed that in asthmatics resistant to steroid treatment, there was an MMP-9/TIMP-1 imbalance that promoted proteolysis and contributed to the chronic remodeling of the airways and the non-reversibility of the bronchial smooth muscle contraction.

Chakir et al. [3] studied bronchial biopsies of patients with moderate and severe asthma treated with oral corticosteroids for two weeks. They showed that this treatment was unable to reduce type I and II collagen or TGF-ß. In contrast, the administration of beclomethasone in daily doses of 800 mg diminished the deposition of collagen in patients with asthma [16]. Miller et al. [30] demonstrated that corticosteroids inhibit TGF beta1 expression in eosinophils and macrophages. McMillan et al. [28] demonstrated in mice chronically exposed to ovalbumin that budesonide was able to reduce collagen deposition and mucus production by regulating inflammation and TGF beta1 signaling rather than by decreasing TGF beta protein production.

Our evaluation of the importance of oxidative stress also demonstrated an attenuation of the number of iNOS-positive cells and the volume fraction of 8-iso-PGF2α in sensitized animals treated solely with the Rho-kinase inhibitor Y-27632 or with the corticosteroid. When the treatments were combined, all of these parameters diminished, but there was a greater reduction in 8-iso-PGF2α in the distal pulmonary tissue.

It has been clearly demonstrated that iNOS activation contributes to the promotion of peroxynitrite production, which leads to lipid peroxidation and isoprostane (8-iso-PGF2α) generation [31]. The isoprostanes contribute to smooth muscle contraction by acting through the tyrosine kinases and Rho/Rho-kinase, leading to the decreased activity of myosin light-chain phosphatase and increasing the level of phosphorylated myosin light-chain and contraction [19].

McGown et al. [27] demonstrated that Fasudil reduced LPS-induced iNOS superregulation, reducing microvascular inflammation. Jiang and George [20] demonstrated that Rho-kinase inhibition with Y-27632 prevented the reduction of NO induced by TGF-β2, thus avoiding iNOS inhibition, and suggesting that TGF-β2 inhibits iNOS expression via a Rho-kinase-dependent pathway in pulmonary epithelial cells.

We observed that the cellular expression of NF-κB was reduced in animals treated with the Rho-kinase inhibitor or with the corticosteroid in sensitized animals. The combination of these treatments allowed for a maximized reduction of NF-κB in the distal pulmonary tissue.

The reduction of NF-κB**-**induced transcription might result in an inhibition of RhoA upregulation induced by IL-13 and TNF-α. Meyer-Schwesinger et al. [30] observed that Rho-kinase inhibition attenuated NF-κB expression, resulting in protection against injury. These data suggest that NF-κB expression may also be Rho-kinase-dependent.

## Conclusions

The data suggest that Rho-kinase inhibition or treatment with corticosteroids can control the pulmonary mechanical response, Th1 and Th2 lymphocyte and eosinophil responses, extracellular matrix remodeling, and the production of oxidative stress in the airways and distal parenchyma in this animal model. Combined treatment with the Rho-kinase inhibitor and the corticosteroid maximized the control of the remodeling response and inflammation in the airways and parenchyma. Rho-kinase inhibition alone or in combination with corticosteroids can be considered a future pharmacological tool for the treatment of chronic pulmonary diseases.

## Abbreviations

AMP: Adenosine monophosphate
AW: Alveolar wall
BVW: Blood vessel wall
BW: Bronchial wall
cAMP: Cyclic adenosine monophosphate
Ca^2+^: Ion calcium
E_NO_: Exhaled nitric oxide
Ers: Elastance of respiratory system
Et: Lung tissue elastance
IFN-γ: Interferon gamma
IgE: Immunoglobulin E
IgG1: Immunoglobulin G1
IL: Interleukin
iNOS: Inducible nitric oxide synthase
LPS: Lipopolysaccharide
MMP: Matrix metalloproteinase
mRNA: Messenger RNA
NF-κB: Nuclear factor kappa B
NO: Nitric oxide
ORC: Animals that received ovalbumin, Y-27632 after the fifth inhalation, and corticosteroid treatment
OVA: Animals that received ovalbumin
OVA-C: Animals that received ovalbumin and corticosteroids
OVA-RHO: Animals that received ovalbumin and Y-27632 after the fifth inhalation exposure
PCA: Passive cutaneous anaphylaxis technique
ROS: Reactive oxygen species
Rrs: Resistance of respiratory system
Rt: Lung tissue resistance
SAL: Animals that received normal saline
TGF-β: Transforming growth factor beta
Th: T helper
TIMP-1: Tissue inhibitor of metalloproteinases-1
Y-27632: (a pyridine derivative, (+)-(R)-trans–4-(1-aminoethyl)-N-(4-pyridyl) cyclohexane carboxamide)
8-iso-PGF2α: 8-iso-prostaglandin F2alpha
